# Effect of Central Injection of Anandamide on LPS-Dependent Suppression of GnRH/LH Secretion in Ewes During the Follicular Phase of the Estrous Cycle

**DOI:** 10.3390/ijms262311246

**Published:** 2025-11-21

**Authors:** Karolina Wojtulewicz, Dorota Tomaszewska-Zaremba, Monika Tomczyk, Joanna Bochenek, Andrzej P. Herman

**Affiliations:** The Kielanowski Institute of Animal Physiology and Nutrition, Polish Academy of Sciences, 05-110 Jabłonna, Poland; k.wojtulewicz@ifzz.pl (K.W.); m.tomczyk@ifzz.pl (M.T.);

**Keywords:** anandamide, endocannabinoids, GnRH, LPS, inflammation, inflammatory cytokines, reproduction

## Abstract

The study investigated the effects of intracerebroventricular (ICV) administration of the endocannabinoid anandamide (AEA) on suppression of gonadotropin-releasing hormone (GnRH)/luteinizing hormone (LH) secretion during lipopolysaccharide (LPS)-induced inflammation in ewes at the follicular phase of the estrous cycle. Animals were divided into three groups: control, LPS (intravenous, IV; 400 ng/kg), and LPS + AEA (ICV; 100 µM/animal). In LPS-treated ewes, AEA increased GnRH concentration in the preoptic area (POA) and upregulated GnRH mRNA expression in the POA and anterior hypothalamus (AHA). Central administration of AEA decreased the circulating concentration of cortisol in LPS-treated ewes. Moreover, AEA lowered proinflammatory interleukin (IL)-1β and increased anti-inflammatory IL-10 protein expressions in the hypothalamus of LPS-treated ewes. However, ICV AEA did not reverse the inflammation-associated reduction in LH secretion. These findings show that acute central administration of AEA abolishes the inhibitory effect of inflammation on GnRH synthesis in the POA and even stimulates it, likely through attenuation of central inflammation, as reflected by IL-1β and IL-10 changes in the POA. Nevertheless, short-term AEA administration was insufficient to counteract the inflammation-mediated suppression of LH secretion. Further studies are needed to explore the role of endocannabinoids (ECBs) in modulating GnRH/LH secretion under inflammatory conditions, particularly with prolonged exposure.

## 1. Introduction

Mammalian reproductive processes are regulated by the hypothalamic-pituitary-gonadal axis (HPG), whose function is highly sensitive to inflammatory processes that can impair fertility [1]. Numerous proinflammatory mediators can disrupt hormonal balance in the reproductive system, and inflammation caused by viral or bacterial infection has been shown to alter hypothalamic and pituitary secretory activity, causing endometriosis and impairing ovarian follicle development [2,3]. Laboratory studies using the bacterial endotoxin lipopolysaccharide (LPS) as an inflammatory factor have demonstrated inhibition of luteinizing hormone (LH) secretion in many animal species, including rats, sheep, cattle, and non-human primates [4,5,6]. This inflammation-induced inhibition of LH secretion is largely attributed to a disruption in the pulsatile release of gonadotropin-releasing hormone (GnRH) from the hypothalamus [4,7].

Proinflammatory cytokines are key mediators of inflammation effects on the HPG axis. Our previous studies have demonstrated that interleukin (IL)-1β reduces GnRH-stimulated LH secretion from anterior pituitary (AP) explants [8], while IL-6 stimulates LH release from pituitary explants [9]. At the pituitary level, the secretory activity of AP cells can be modulated not only by blood-borne cytokines acting through their corresponding receptors on pituitary cells but also via paracrine mechanisms involving proinflammatory cytokines synthesized locally in the AP during inflammation [10]. At the hypothalamic level, the inhibitory effects of proinflammatory cytokines on GnRH secretion may occur through direct action on GnRH neurons or indirectly via mediators such as opioids, catecholamines, gamma-aminobutyric acid, prostaglandins, or nitric oxide [11].

Cannabis plants have been used for medicinal purposes since ancient times [12]. Their use has become particularly popular among young people aged 18–30, i.e., during the period of greatest reproductive activity [13,14]. Nevertheless, research on the effects of cannabinoids (CBs) on reproduction remains inconclusive. Hemp contains over one hundred distinct phytocannabinoids, including the well-known compounds delta-9-tetrahydrocannabinol (THC) and cannabidiol (CBD), along with a wide range of terpenoids, flavonoids, and alkaloids [15,16]. Cannabinoids are also synthesized by animals and activate cannabinoid receptors expressed in many tissues. Anandamide (AEA), the first identified and most studied endocannabinoid, is a fatty acyl lipid found in the brains of pigs [17], rats, mice, mouse spinal cords, human hippocampi, and other mammalian tissues [18]. AEA binds to cannabinoid receptors type 1 (CB1R) and 2 (CB2R), acts as a partial agonist of both, and also interacts with non-cannabinoid targets such as vanilloid receptors [18,19]. CB1Rs are mainly expressed in the central nervous system, spinal cord, endocrine organs, ovaries, endometrium, and testes [20,21]. CB2Rs have primarily immunomodulatory and anti-inflammatory roles and are localized in the peripheral nervous system, immune cells, and ovarian structures such as the cortex, medulla, and follicles [22]. The endocannabinoid (ECB) system has been identified in hypothalamic areas involved in hormone production, including GnRH, and through its interaction with the hypothalamic-pituitary-ovarian axis, it participates in the control of reproductive processes [23]. AEA secretion is regulated by hormonal signals [24]: estrogen from ovarian follicles indirectly increases AEA levels, while postovulatory inhibition of estrogen secretion causes AEA degradation [25]. The specific role of AEA in modulating GnRH secretion, however, is still elusive. CB1R stimulation in the hypothalamus has been shown to reduce the release of GnRH, leading to lower secretion of follicle-stimulating hormone (FSH) and LH in the pituitary, consequently suppressing gonadal function and estrogen and progesterone production [25]. In contrast, our previous studies in ewes suggested a stimulatory effect of centrally administered AEA on hypothalamic GnRH synthesis, an effect that may be partially mediated by central prostaglandins [26].

Exogenous, endogenous, and synthetic CBs are widely recognized as regulators of the immune system. Their effects on immune cells can be either stimulatory or suppressive, depending on the receptor type, concentration, and target cell population. Both types of cannabinoid receptors are present on immune cells, and their expression levels are modulated by physiological conditions such as infection or immune activation. In the central nervous system (CNS), microglia express both receptor subtypes, with CB2R markedly upregulated during microglial activation (reviewed in [27,28]). Evidence suggests that CB2Rs regulate microglial activity by modulating the NF-κB signaling pathway as well as mitogen-activated protein kinase (MAPK) pathways, including c-Jun N-terminal kinase (JNK), ERK, and p38. These kinases are recognized as key modulators of inflammatory processes, as they regulate downstream signaling in the innate immune response and the production of inflammatory mediators [29].

Although there are reports concerning the influence of CBs on the reproductive system as well as the interaction between ECBs and immunological systems, there is a lack of comprehensive research on the role of CBs in the regulation of GnRH/LH secretion during an inflammatory state induced by bacterial or viral infections or in autoimmunological diseases. It is well established that cannabinoids affect the female reproductive system, causing disturbances in the secretion of HPG axis hormones. There are also many studies concerning the influence of CBs on the immunological system activity. However, the problem of how ECBs affect the reproductive system during immune stress remains unstudied. Considering that central AEA can stimulate GnRH synthesis in sheep and modulate the production of inflammatory mediators, we investigated the effects of intra-cerebroventricular (ICV) AEA administration on the LPS-dependent suppression of GnRH/LH secretion in ewes during the follicular phase.

## 2. Results

### 2.1. Blood LH and Cortisol Concentrations

**Figure 1:**
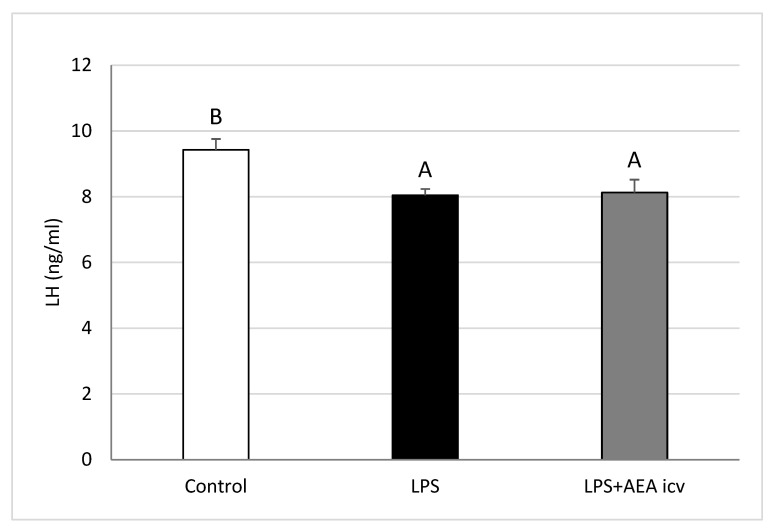
Effect of lipopolysaccharide (LPS; 400 ng/kg, IV) and anandamide (AEA; 100 µM/animal, ICV) injections on plasma luteinizing hormone (LH) concentrations. Data are expressed as mean ± SEM (*n* = 6 animals per group) for hormone concentrations measured 2–3 h after LPS treatment. Significant differences are indicated by different capital letters (one-way ANOVA followed by Tukey’s post hoc test). Statistical significance was accepted at *p* < 0.05 (Control vs. LPS *p* = 0.018988; Control vs. LPS + AEA ICV *p* = 0.027471).

Systemic inflammation significantly decreased (*p* ≤ 0.05) LH levels in sheep. However, ICV AEA administration did not reverse this inflammation-induced suppression of LH secretion (Figure 1).

**Figure 2:**
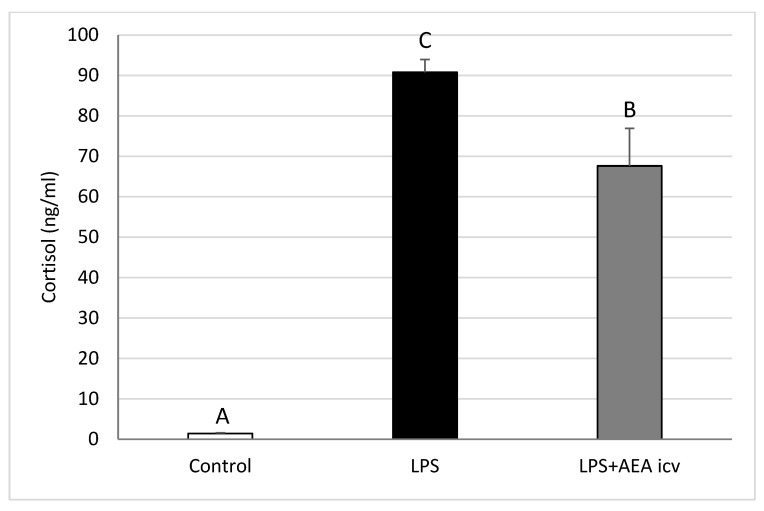
Effect of lipopolysaccharide (LPS; 400 ng/kg, IV) and anandamide (AEA; 100 µM/animal, ICV) injections on plasma cortisol concentrations. Data are expressed as mean ± SEM (*n* = 6 animals per group) for hormone concentrations measured 2–3 h after LPS treatment. Significant differences are indicated by different capital letters (one-way ANOVA followed by Tukey’s post hoc test). Statistical significance was accepted at *p* < 0.05 (Control vs. LPS *p* = 0.000178; Control vs. LPS + AEA ICV *p* = 0.000178; LPS vs. LPS + AEA ICV *p* = 0.028261).

Blood cortisol levels were significantly elevated (*p* ≤ 0.05) in LPS-treated animals compared to the control group. AEA ICV decreased (*p* ≤ 0.05) cortisol levels in sheep with induced inflammation; however, circulating cortisol concentration remained elevated (*p* ≤ 0.05) compared to control animals (Figure 2).

### 2.2. GnRH and Cytokine Levels in the POA

**Figure 3:**
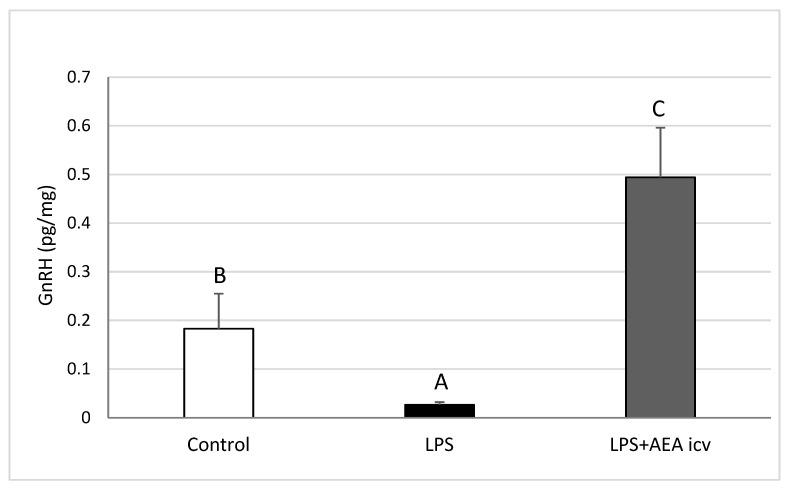
Effect of lipopolysaccharide (LPS; 100 ng/kg, IV) and anandamide (AEA; 100 µM/animal, ICV) injections on gonadotropin-releasing hormone (GnRH) concentration in the preoptic area (POA). Data are expressed as mean ± SEM (*n* = 6 animals per group). Significant differences are indicated by different capital letters (one-way ANOVA followed by Tukey’s post hoc test). Statistical significance was accepted at *p* < 0.05. (Control vs. LPS *p* = 0.006320; Control vs. LPS + AEA ICV *p* = 0.029453; LPS vs. LPS + AEA ICV *p* = 0.000194).

Induction of inflammation resulted in a reduction (*p* ≤ 0.01) of the content of GnRH in the preoptic area (POA). AEA administration during inflammation not only reversed this suppression but even increased GnRH levels to values significantly higher (*p* ≤ 0.05) than those in the control group (Figure 3).

**Figure 4:**
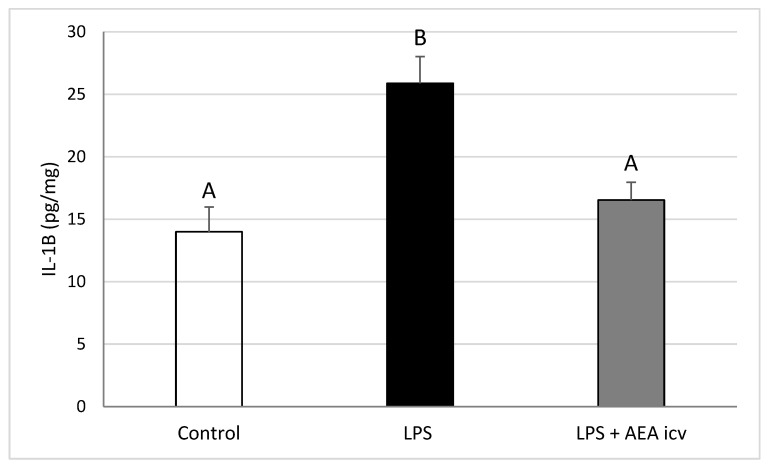
Effect of lipopolysaccharide (LPS; 100 ng/kg, IV) and anandamide (AEA; 100 µM/animal, ICV) injections on Interleukin -1β (IL-1β) concentration in the preoptic area (POA). Data are presented as mean ± S.E.M. (*n* = 6 animals per group). Significant differences are indicated by different capital letters (one-way ANOVA followed by Tukey’s post hoc test). Statistical significance was stated when *p* < 0.05 (Control vs. LPS *p* = 0.001097; LPS vs. LPS + AEA ICV *p* = 0.005727).

**Figure 5:**
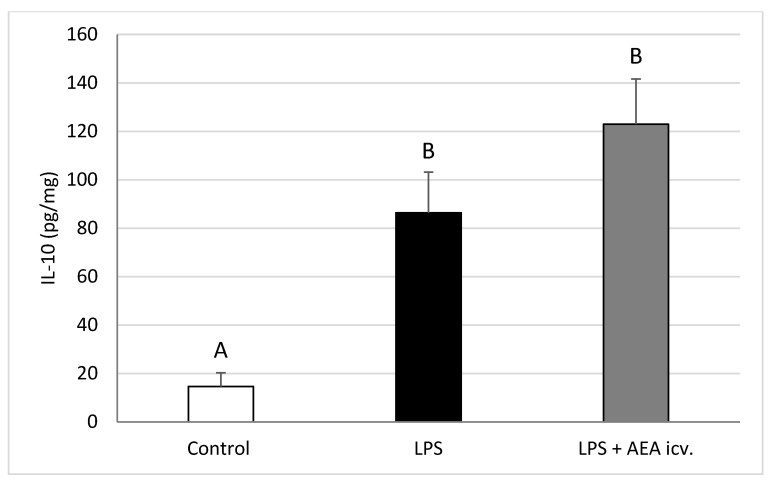
Effect of lipopolysaccharide (LPS; 100 ng/kg, IV) and anandamide (AEA; 100 µM/animal, ICV) injections on interleukin-10 (IL-10) concentration in the preoptic area (POA). Data are expressed as mean ± SEM (*n* = 6 animals per group). Significant differences are indicated by different capital letters (one-way ANOVA followed by Tukey’s post hoc test). Statistical significance was accepted at *p* < 0.05. (Control vs. LPS *p* = 0.001680; Control vs. LPS + AEA ICV *p* = 0.000193).

Interleukin-1β levels in the POA were elevated (*p* ≤ 0.01) by LPS-induced inflammation, but ICV AEA administration eliminated (*p* ≤ 0.01) this stimulatory effect, restoring IL1B levels to control values (Figure 4). Endotoxin treatment also increased (*p* ≤ 0.01) IL-10 concentration in the POA, whereas central AEA administration during inflammation further raised (*p* ≤ 0.01) IL-10 levels compared with both the control (*p* ≤ 0.001) and LPS-only (*p* ≤ 0.05) groups (Figure 5).

### 2.3. GnRH and GnRHR Gene Expression in the Hypothalamus

**Figure 6:**
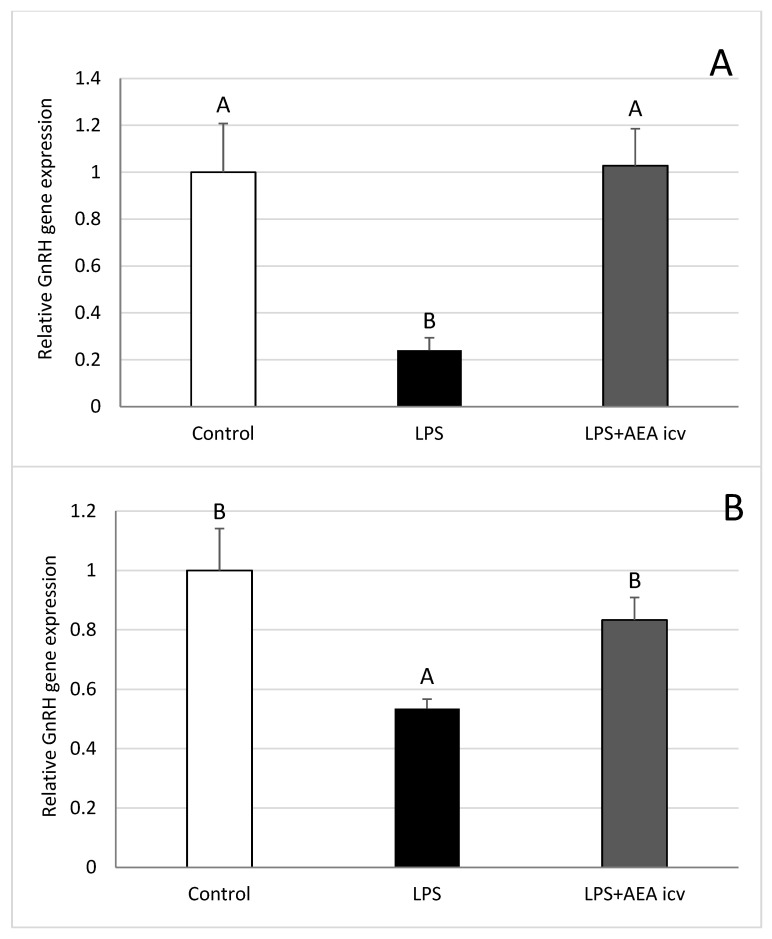

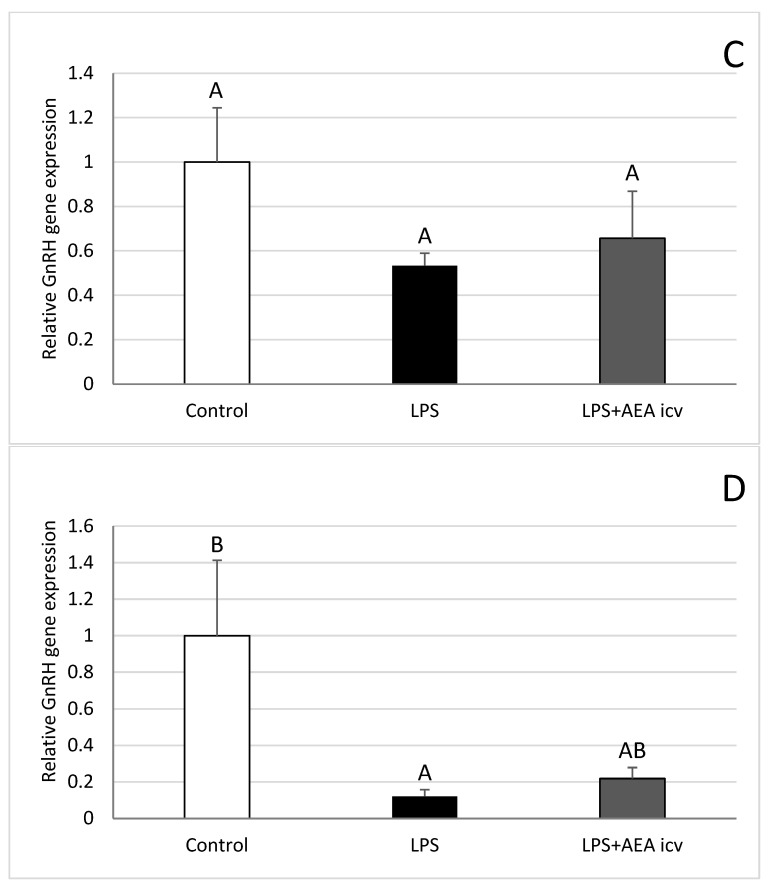
Effect of lipopolysaccharide (LPS; 400 ng/kg, IV) and anandamide (AEA; 100 µM/animal, ICV) injections on relative gonadotropin releasing hormone (GnRH) gene expression in the preoptic area (POA) (**A**), anterior hypothalamus (AHA) (**B**), mediobasal hypothalamus (MBH) (**C**), and median eminence (ME) (**D**). Data are expressed as mean ± SEM (*n* = 6 animals per group). Significant differences are indicated by different capital letters (one-way ANOVA followed by Tukey’s post hoc test). Statistical significance was accepted at *p* < 0.05. (POA: Control vs. LPS *p* = 0.003885; Control vs. LPS + AEA ICV *p* = 0.002940; AHA: Control vs. LPS *p* = 0.010088; Control vs. LPS + AEA ICV *p* = 0.049125; ME: Control vs. LPS *p* = 0.048266).

Inflammation induced by IV administration of bacterial endotoxin reduced (*p* ≤ 0.05) GnRH gene expression in the POA (Figure 6A), anterior hypothalamus (AHA) (Figure 6B), and median eminence (ME) (Figure 6D). Simultaneous ICV administration of AEA abolished the inhibitory effect of LPS on GnRH gene expression in the POA (Figure 6A) and AHA (Figure 6B). No beneficial effect of AEA was observed in the ME during inflammation (Figure 6D). GnRH gene expression in the mediobasal hypothalamus (MBH) was not significantly affected by either LPS or AEA administration (Figure 6C).

**Figure 7:**
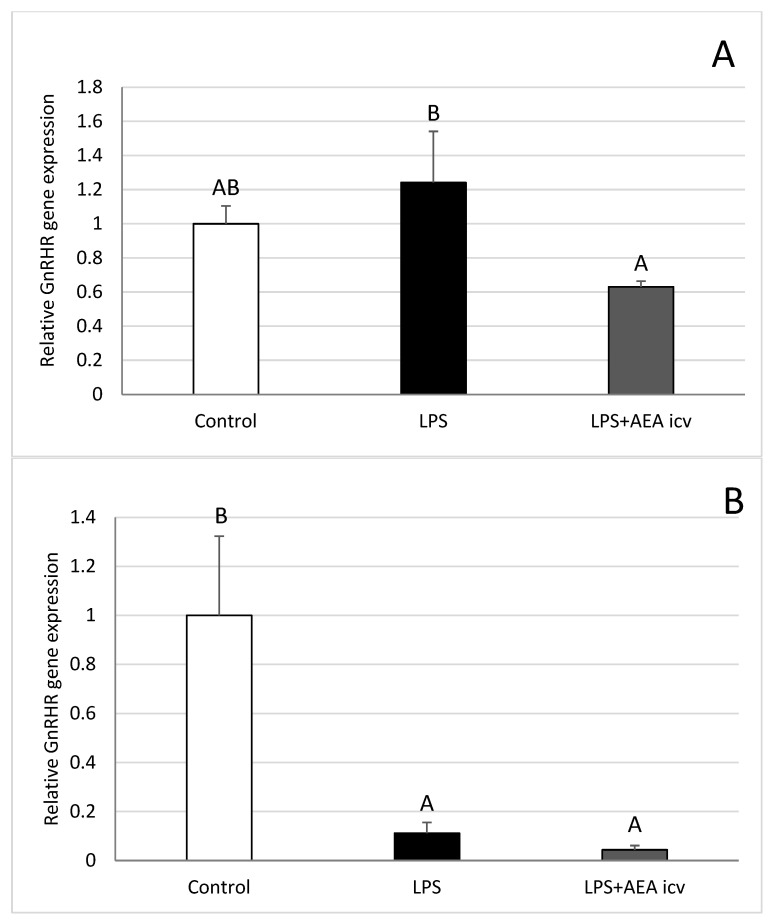

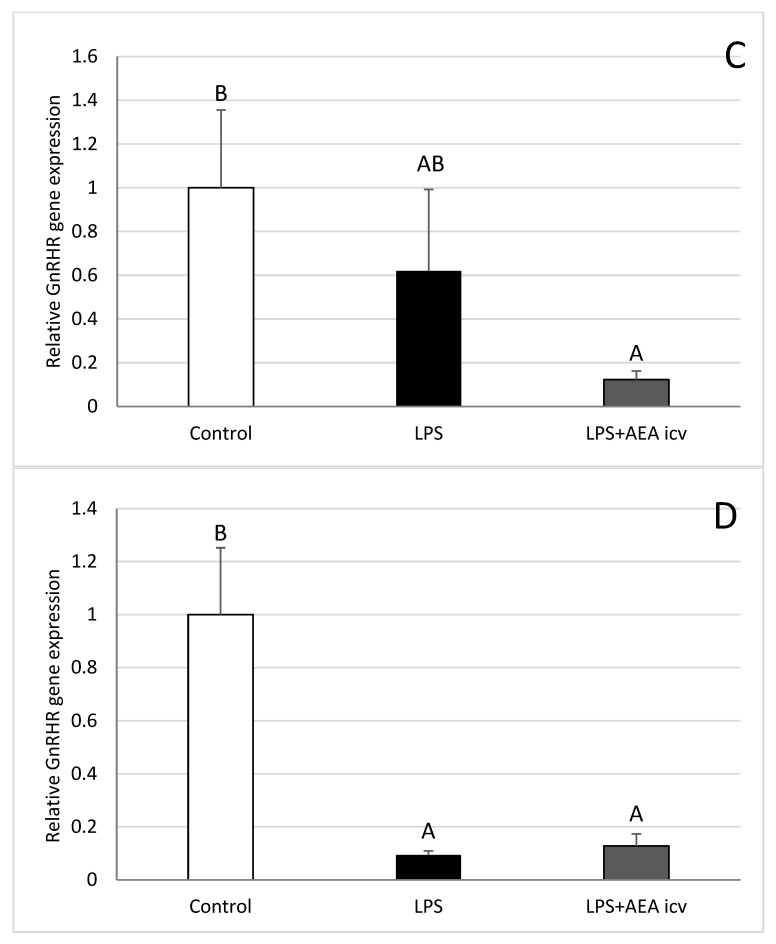
Effect of lipopolysaccharide (LPS; 400 ng/kg, IV) and anandamide (AEA; 100 µM/animal, ICV) injections on relative gonadotropin-releasing hormone receptor (GnRHR) gene expression in the preoptic area (POA) (**A**), anterior hypothalamus (AHA) (**B**), mediobasal hypothalamus (MBH) (**C**), and median eminence (ME) (**D**). Data are expressed as mean ± SEM (*n* = 6 animals per group). Significant differences are indicated by different capital letters (one-way ANOVA followed by Tukey’s post hoc test. Statistical significance was accepted at *p* < 0.05. (POA: LPS vs. LPS + AEA ICV *p* = 0.036790; AHA: Control vs. LPS *p* = 0.011819; Control vs. LPS + AEA ICV *p* = 0.007191; MBH: Control vs. LPS + AEA ICV *p* = 0.049222; ME: Control vs. LPS *p* = 0.000682; Control vs. LPS + AEA ICV *p* = 0.000957).

Administration of bacterial endotoxin reduced (*p* ≤ 0.05) GnRH receptor (GnRHR) gene expression in the AHA (Figure 7B) and ME (Figure 7D), while ICV AEA administration did not alter the inhibitory effect of LPS in these structures. In the MBH (Figure 7C), endotoxin alone did not affect GnRHR gene expression; however, the expression level of this gene was reduced (*p* ≤ 0.05) in animals administered AEA compared with the control group. On the other hand, analysis of GnRHR gene expression in the POA (Figure 7A) showed that AEA treatment reduced (*p* ≤ 0.05) this gene expression compared with only LPS-treated ewes.

### 2.4. GnRHR and LH Gene Expression in AP

**Figure 8:**
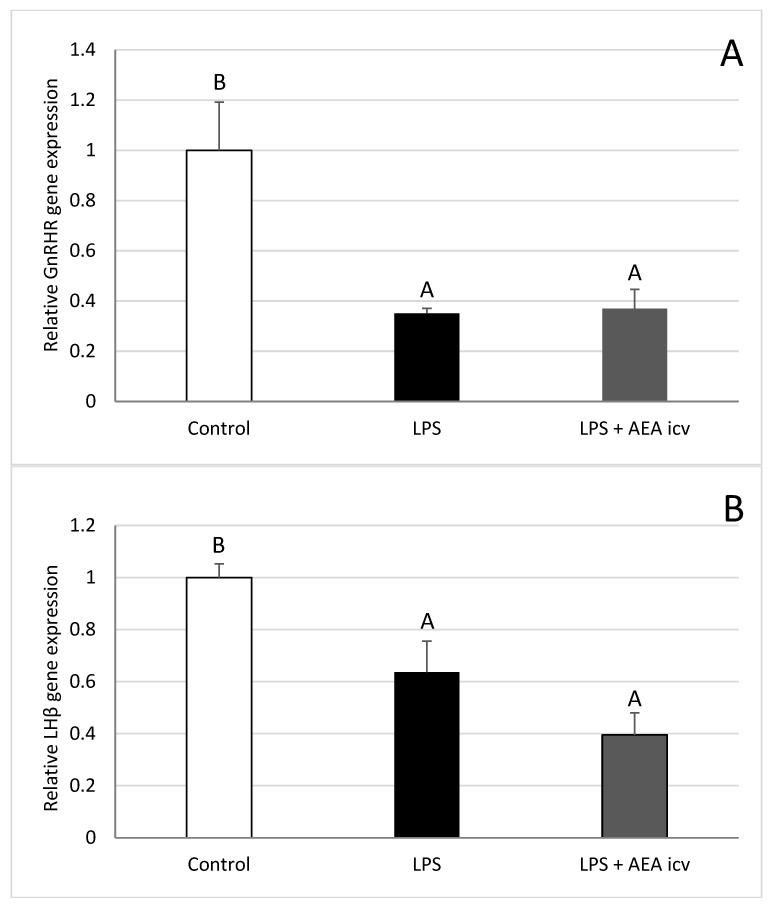
Effect of lipopolysaccharide (LPS; 400 ng/kg, IV) and anandamide (AEA; 100 µM/animal, ICV) injections on relative gonadotropin-releasing hormone receptor (GnRHR) (**A**) and LHβ (**B**) gene expression in the anterior pituitary (AP). Data are expressed as mean ± SEM (*n* = 6 animals per group). Significant differences are indicated by different capital letters (one-way ANOVA followed by Tukey’s post hoc test). Statistical significance was accepted at *p* < 0.05. (GnRHR: Control vs. LPS *p* = 0.004436; Control vs. LPS + AEA ICV *p* = 0.005600; LHβ: Control vs. LPS *p* = 0.014081; Control vs. LPS + AEA ICV *p* = 0.000395).

Inflammation induced by IV LPS administration reduced (*p* ≤ 0.05) GnRHR (Figure 8A) and LHβ (Figure 8B) gene expression in the ovine AP. AEA administration did not affect GnRHR or LH gene expression in LPS-treated ewes.

## 3. Discussion

The present study demonstrates that AEA effectively reverses the inhibitory effects of LPS on GnRH secretion in ewes during the follicular phase of the estrous cycle. Intracerebroventricular administration of LPS at a dose of 400 ng/kg decreased GnRH gene expression in the preoptic area (POA) and the median eminence (ME). A reduction in GnRH peptide expression was also observed in the POA. These findings are consistent with our previous study, which showed that LPS suppresses hypothalamic GnRH secretion [30]. In our earlier work on anestrous ewes, endotoxin-dependent inflammation reduced GnRH mRNA expression, particularly in the POA, the hypothalamic structure where the majority of GnRH neurons are located [7]. The inhibitory effect of LPS-induced inflammation on GnRH synthesis is likely mediated by centrally acting proinflammatory cytokines, particularly IL-1β and TNFα [31,32]. These cytokines may be mainly of peripheral origin [33,34], although local synthesis within the hypothalamus is an additional important source [35]. Microglial cells are considered the primary site of proinflammatory cytokine synthesis within the central nervous system [36]. Although their role in disrupting hypothalamic secretory activity during endotoxin-induced inflammation seems to be unquestionable, changes in GnRH secretion may also be caused by the direct central action of LPS [7], as its molecules are partially able to cross the blood–brain barrier (BBB). Studies in rats [37] and mice [38] demonstrated that measurable amounts of iodine-radiolabeled LPS cross the BBB and reach the brain parenchyma. Evidence from rat studies also implies that LPS enters the brain under normal physiological circumstances, possibly through transport involving lipoproteins [38].

In the present experiment, we demonstrated that central administration of AEA prevented LPS-induced suppression of GnRH secretion in ewes during the follicular phase. This effect may result from a direct action of AEA on GnRH secretion or from its anti-inflammatory properties mediated through the modulation of cytokine activity. Our previous experiment on anestrous ewes showed that ICV injection of AEA at a concentration of 30 µM elevated GnRH mRNA expression in the ME, but not in the POA. However, AEA exerted a stimulatory effect on GnRH peptide content in both the POA and ME [26]. In contrast, studies conducted primarily in rats have shown that cannabinoids (CBs) and endocannabinoids (ECBs) generally exert an inhibitory effect on the HPG axis. Scorticati et al. [39] demonstrated that AEA suppressed GnRH release from the MBH in male rats, while it had no effect on hypothalamic GnRH secretion in ovariectomized (OVX) females. However, in vitro studies using MBH tissue from estrogen-supplemented OVX rats (OVX-E) demonstrated that AEA stimulated GnRH release, whereas no such effect was observed in OVX rats without estrogen administration. These findings suggest that estrogen may reverse the inhibitory effect of AEA on GnRH secretion in female rats. A potential mechanism for this inhibition involves gamma-aminobutyric acid (GABA), a key neurotransmitter regulating GnRH secretion. Fernandes-Solari et al. [40] demonstrated that AEA incubation significantly increased GABA release from the MBH of male rats in vitro. Since CB1Rs are expressed on GABAergic interneurons in the hippocampus and neocortex [23], and GABA generally inhibits GnRH secretion, increased GABA release may contribute to reduced GnRH output. In ewes, GABA(A) receptors also play a significant role in regulating GnRH secretion, although the underlying neural mechanisms may differ. The inhibitory effects of GABA may be mediated through interactions with β-endorphinergic and catecholaminergic systems, whose activity depends on the animal’s physiological state [41]. Cannabinoids have been shown to influence catecholaminergic and serotonergic systems, both of which are involved in the hypothalamic regulation of GnRH secretion. ICV administration of AEA has been shown to increase hypothalamic norepinephrine levels in mice [42]. Given the significant role of the dopaminergic system in regulating GnRH secretion in ewes [43], its interaction with cannabinoids is of particular relevance. Scorticati et al. [39] showed that cannabinoids can affect brain dopamine (DA) levels and dopamine receptor expression. Their study further demonstrated that AEA stimulates both the synthesis and release of DA from MBH explants, as evidenced by an increased ratio of 3,4-dihydroxyphenylacetic acid (DOPAC) and DA, indicating accelerated DA turnover. Interestingly, the effect of AEA on dopaminergic neuronal activity differed between male and female rats that were either OVX or OVX-E [39]. These findings imply that estrogen may stimulate AEA release from MBH neurons, which then acts on cannabinoid receptors located on tuberoinfundibular DA neurons to inhibit DA release. This hypothesis is supported by the observation that CB1R colocalizes with the cell bodies of periventricular tyrosine hydroxylase-containing neurons, DA neurons that project to the ME, where DA can regulate GnRH secretion [44]. Overall, the impact of AEA on GnRH release appears to stem from complex interplay between the endocannabinoid system and neurotransmitters such as catecholamines.

Unexpectedly, in the present study, ICV administration of AEA did not affect LPS-inhibited LH concentrations. The only effect was that LPS reduced LHβ gene expression in the AP, and this effect was not reversed by ICV administration of AEA. It is important to emphasize that LH was assessed at 2–3 h post—LPS treatment and that pulsatile secretion of this hormone defines feature of LH dynamics. To fully understand changes in LH secretion the serial blood sampling and pulse analysis is needed. Our previous research on follicular-phase ewes demonstrated that LPS suppressed LH secretion [3]. Similarly, inhibitory effects of bacterial endotoxin have been reported in anestrous ewes, where LPS diminished both LHβ gene expression and LH release [45]. Disturbances in LH secretion in the AP accompanying inflammation appear to result mainly from reduced GnRH secretion in the hypothalamus. However, the changes in the secretory activity of the pituitary may also result, at least partially, from the direct action of LPS on this gland [46,47]. Here, central (ICV) administration of AEA did not reverse the LPS-induced decrease in LHβ gene expression in the AP. This contrasts with numerous rat studies demonstrating that both exogenous (e.g., THC) and endogenous (e.g., AEA) cannabinoids inhibit gonadotropin release. For instance, chronic supply of cannabinoids in prepubertal female rats reduced serum LH and sex steroid levels [48], while acute THC treatment inhibited pulsatile LH secretion and blocked estrogen- or estrogen/progesterone-induced LH surge in ovariectomized rats [49]. In our previous in vitro study employing AP explants from anestrous ewes, AEA also directly suppressed LH secretion [50]. However, that model involved direct exposure of AP cells to AEA, while the present in vivo approach allowed AEA to act through integrated pathways at both hypothalamic and pituitary levels. These results may reflect incomplete restoration of HPG axis signaling at the pituitary level. On the one hand, the lack of effect of AEA on LH release during immunological stress may be due to the reduced level of GnRHR gene expression in the AP. We found that LPS-induced inflammation decreased GnRHR gene expression in the ME and AP. AEA had no impact on the LPS-induced reduction in GnRHR gene expression. The above-mentioned studies ex vivo study on ovine AP explants demonstrated that AEA could inhibit LH secretion by downregulating GnRHR [50]. On the other hand, in our study, AEA was administered directly into the hypothalamus region and its anti-inflammatory effect was found in this area. However, the administration of LPS induces a strong inflammatory response, including significantly increasing the level of proinflammatory cytokines circulating in the blood, as shown in animal studies and also in humans [51,52]. Circulating inflammatory mediators can directly affect processes occurring in the pituitary, because this gland sits outside the blood–brain barrier. The study on rodents showed that both posterior and anterior pituitary gland expressed large number of IL-1 receptors and suggested pituitary cells may be a direct target for IL-1 [53]. Our recent findings revealed that the ovine anterior pituitary expresses considerable levels of mRNA for proinflammatory cytokines, including IL-1β, IL-6, and TNFα, along with their respective receptors [10]. The expression of cytokine receptors by pituitary cells suggests that these ligands can modulate the gland’s hormonal secretory functions. In support of this, our previous ex vivo experiment demonstrated that IL-1β inhibited LH secretion and decreased GnRHR gene expression in GnRH-stimulated ovine pituitary explants [8]. Such direct pituitary effects of inflammatory mediators like IL-1β may underlie the lack of LH response despite the AEA-induced stimulation of hypothalamic GnRH secretion.

Another mechanism by which AEA may counteract the LPS-induced suppression of GnRH/LH secretion is through its anti-inflammatory properties, mediated by the modulation of cytokine activity. Exogenous, endogenous, and synthetic cannabinoids are widely recognized as immune regulators [27]. Here, AEA reduced LPS-stimulated IL-1β concentration at the hypothalamic level, although no changes in gene expression were observed. We also found that AEA increased IL-1Ra gene expression in hypothalamic structures. Interestingly, IL-10 concentrations were significantly elevated after AEA treatment compared with control and LPS-treated ewes. These findings suggest that the antiinflammatory action of central AEA may rely on the suppression of proinflammatory mediators such as IL-1β, consistent with previous studies showing similar effects following peripheral AEA administration during endotoxin-induced inflammation [54]. Additionally, AEA treatment has been shown to reduce serum levels of the proinflammatory cytokine IL-1β in rats subjected to water immersion and restraint stress [55]. Cannabinoids also display anti-inflammatory properties in the brain in neurodegenerative diseases associated with neuroinflammation. In murine neurodegeneration models, cannabidiol has been shown to decrease CNS levels of proinflammatory cytokines, including IL-1β [56]. Similarly, in virus-infected mice, R(+)WIN55,212, a CB1 receptor agonist, significantly reduced mRNA expression of IL-1β, IL-6, and TNFα [57]. The anti-inflammatory action of central AEA observed in our study likely involves a dual mechanism: the suppression of proinflammatory mediators and the stimulation of anti-inflammatory cytokines like IL-10. This aligns with in vitro studies demonstrating that AEA promotes IL-10 production in microglial cells, primarily through CB2 receptor activation and downstream anti-inflammatory signaling pathways [58]. Therefore, the significant increase in IL-10 concentration following AEA treatment in our experiment is consistent with this established mechanism. Correa et al. [58] have demonstrated that AEA increases LPS/IFN-γ-induced IL-10 production in microglia by targeting CB2Rs and downstream ERK1/2 and JNK MAPKs signaling pathways. The relationship between cannabinoids and cytokines is bidirectional, as they can influence cytokine secretion, while cytokines can modulate cannabinoid receptor activity. This dynamic interplay has the potential to alter cytokine profiles from a proinflammatory to an anti-inflammatory state [59]. Stimulation of CB1R by synthetic cannabinoids suppresses the synthesis of proinflammatory cytokines in glial cells, demonstrating anti-inflammatory effects in vitro [60,61]. It should be emphasized that research conducted on a mouse model with CB1R deletion specifically in microglia, subjected to an immune challenge, indicated that the role of CB1R in regulating neuroinflammation is more complex than previously thought. The study revealed that microglial CB1R differentially modulates sickness behavior in males and females. In males, knocking out CB1R in CX3CR1-positive cells reduced IL-1β and TNF-α mRNA synthesis, whereas in females, microglial CB1R deletion had no significant effect on cytokine production in the hippocampus [62]. In our in vivo experiment we did not use CB1R and CB2R antagonists, so we cannot infer the role of CB1R vs. CB2R. Further studies with agonists and antagonists of individual receptors are required. Conversely, endocannabinoids such as AEA can serve as a source of arachidonic acid (AA), which is further metabolized by eicosanoid-synthesizing enzymes to generate bioactive lipids possessing pro- or anti-inflammatory functions. Pharmacological blockade of endocannabinoid degradation may increase the levels of these immunomodulatory lipids, consequently affecting the activity of inflammatory cells [63]. Arachidonic acid (AA) is a polyunsaturated fatty acid found in the phospholipids of cell membranes throughout the body and is particularly abundant in the brain. Through enzymatic conversion of AA, mediators such as prostaglandins, thromboxanes, and leukotrienes are synthesized de novo. One of these products is prostaglandin E_2_ (PGE_2_). In both the pituitary and hypothalamus, AA and its metabolites play important roles in regulating the secretion of various peptide hormones [64]. A few studies have demonstrated that central administration of the phospholipase A_2_ activator melittin, arachidonic acid (AA), or thromboxane A_2_ can stimulate the catecholaminergic, vasopressinergic, and renin–angiotensin systems [65,66]. These neuropeptides are capable of modulating GnRH and LH secretion. Moreover, numerous studies have demonstrated that PGE_2_ serves as an important regulator of GnRH release [67]. Additionally, intracerebroventricular administration of AA was shown to elevate plasma FSH, LH, and testosterone concentrations in conscious male rats [68]. Additionally, the anti-inflammatory effect of AEA in the CNS may involve increased expression of IL-1Ra and IL-1R2 genes [69].

It is worth mentioning that our study showed that LPS treatment significantly elevated the circulating level of cortisol, whereas this stimulatory effect had been reduced in AEA-treated animals. An increase in circulating glucocorticoids during stress is thought to inhibit the secretion of GnRH and gonadotropins. In sheep, cortisol has been shown to act at the pituitary level to decrease its responsiveness to GnRH and to lower LH pulse frequency. Moreover, cortisol suppresses GnRH pulse frequency in follicular-phase ewes, an effect that appears to depend on the presence of ovarian steroids [70]. Nonetheless, accumulating evidence suggests that the impact of stress on gonadotropin secretion is not straightforward. Recent findings indicate that acute stress may activate rather than inhibit the hypothalamic–pituitary system. In rats, acute stress has been shown to accelerate and intensify the preovulatory LH surge [71]. It has been proposed that activation of corticotropin-releasing hormone (CRH) receptors, particularly CRHR1, may enhance LH secretion under specific conditions. Endogenous cannabinoids are key regulators of both the physiological and behavioral aspects of stress responses. It is also considered that through buffering basal activity and mediating fast glucocorticoid feedback, endocannabinoid signaling contributes to the maintenance of HPA axis homeostasis [72]; therefore, decreased circulating cortisol concentration after AEA treatment is not surprising. We cannot exclude that activation of the HPA axis and interaction of AEA with glucocorticoid secretion influenced GnRH/LH secretion, but this requires further in-depth research.

## 4. Materials and Methods

### 4.1. Animals and Experimental Procedures

The experiment was conducted in November using mature female Blackface sheep, and maintained under good husbandry conditions. The animals were acclimated to the experimental environment for one month before the study. They were fed ad libitum according to the recommendations of the National Research Institute of Animal Production for adult ewes [73]. To minimize isolation stress, the sheep had constant visual contact with each other.

One month before the experiment, all ewes (*n* = 18) underwent the surgical implantation of a cannula into the third brain ventricle. During surgery, all animals were anesthetized with infusion drugs, and their vital parameters were constantly monitored. Postoperatively, analgesics and antibiotics were administered for three days to prevent complications. The animals’ health status was closely monitored until complete recovery. After a one-month convalescence period, the estrous cycles of the ewes were synchronized to standardize experimental conditions using Chronogest CR sponges (MSD Animal Health, Walton, UK), following the procedure described by Przybył et al. [74]. Each ewe received one intravaginal polyester–polyurethane sponge containing 20 mg of flugestone acetate (a synthetic progesterone analog), irrespective of body weight or the stage of the estrous cycle. After 14 days, the sponges were carefully removed, and pregnant mare serum gonadotropin (PMSG; 500 IU per animal; Folligon, Intervet Int., Boxmeer, The Netherlands) was administered intramuscularly. The experimental procedure was performed 24 h following PMSG injection in the follicular phase of the estrous cycle.

Venous catheters were implanted into the jugular vein one day before the experiment. The animals (*n* = 18) were randomly allocated to three groups, as presented in Table 1. Immune stress was induced by intravenous (IV) administration of lipopolysaccharide (LPS; Escherichia coli 055:B5, Merck KGaA, Darmstadt, Germany) at a dose of 400 ng/kg. This procedure, which serves as a standard method for eliciting systemic inflammation in sheep, has been applied in several of our previous studies and by other researchers [1,7,75]. The LPS was dissolved in 0.9% (*w*/*v*) saline (Baxter, Deerfield, IL, USA) to a final concentration of 10 mg/L. Jugular blood samples (10 mL) were collected at 15 min intervals, starting 2 h before IV administration of LPS or an equivalent volume of saline, and continued for 3 h afterward. Two hours after LPS/saline treatment, the animals were administered an intracerebroventricular (ICV) injection of AEA (100 µM/animal) or an equivalent volume of Ringer–Locke solution into the third brain ventricle (Group 3). The AEA dose was selected based on a preliminary experiment testing concentrations of 1, 10, 30, and 100 µM, in which 100 µM was determined to be optimal with respect to LH release and animal behavior. After blood collection, the animals were immediately euthanized (3 h after LPS or saline administration), and the brains were rapidly removed. Hypothalamic structures, including the POA, AHA, MBH, and ME, were dissected according to a sheep brain stereotaxic atlas [76]. The mammillary body, ME, and optic chiasm were used as landmarks. Cuts were made to a depth of 2 to 2.5 mm for the MBH and 2.5 to 3 mm for the AHA and POA. All collected tissues were immediately frozen in liquid nitrogen and stored at −80 °C.

### 4.2. Assays

#### 4.2.1. Radioimmunoassay for LH, FSH, and Cortisol

The concentration of gonadotropins and cortisol in blood plasma was measured using a double-antibody radioimmunoassay (RIA) according to the protocol described in Herman et al. [1]

#### 4.2.2. ELISA for GnRH Concentration in the POA

GnRH concentrations in POA homogenates, were determined using commercial ELISA kits: GnRH ELISA kit (Cat. No. ESH0062, FineTest Biotech Inc., Boulder, CO, USA), IL-1β ELISA kit (Cat. No. ESH0012; Wuhan Fine Biotech Co., Ltd., Wuhan, China) and IL-10 ELISA kit (Cat. No. ESH0009; Wuhan Fine Biotech Co., Ltd., Wuhan, China), all validated for sheep. Assays were performed according to the manufacturer’s instructions. The plates were incubated, and absorbance was measured at 450 nm using a VersaMax microplate reader (Molecular Devices LLC, Sunnyvale, CA, USA). GnRH, IL-1β, and TNFα concentrations were normalized to the total protein content of each sample, as determined by the Bradford method.

#### 4.2.3. Relative Gene Expression

Total RNA was extracted from the hypothalamus and anterior pituitary using the NucleoSpin^®^ RNA/Protein Kit (MACHEREY-NAGEL GmbH & Co., Düren, Germany) in accordance with the manufacturer’s guidelines. The purity and concentration of isolated RNA were quantified spectrophotometrically by measuring the optical density at 230, 260, and 280 nm in a NanoDrop 1000 instrument (Thermo Fisher Scientific Inc., Waltham, MA, USA). The RNA integrity was verified by electrophoresis using a 1.2% agarose gel stained with ethidium bromide (Merck KGaA, Darmstadt, Germany). Maxima™ First Strand cDNA Synthesis Kit for RT-qPCR (Thermo Fisher Scientific Inc., Waltham, MA, USA) was used to prepare cDNA synthesis. As a starting material for this PCR synthesis 800 ng of total RNA was used.

Real-time RT-PCR was conducted using HOT FIREPol^®^ EvaGreen^®^ qPCR Mix Plus (Solis BioDyne, Tartu, Estonia) and HPLC-purified primers from Genomed (Warszawa, Poland), as described previously [35]. Specific primers for housekeeping genes and target genes were selected based on previous studies (Table 2). Amplification was carried out on a Rotor-Gene 6000 (Qiagen, Duesseldorf, Germany) under the following conditions: initial activation at 95 °C for 15 min; 35 cycles of denaturation at 94 °C for 5 s, 5 annealing at 59 °C for 20 s, and extension at 72 °C for 5 s. A final melting-curve analysis with continuous fluorescence measurement was performed to verify amplification specificity.

Relative gene expression was calculated using the comparative quantification option [77] in Rotor Gene 6000 software version 1.7 (Qiagen, Dusseldorf, Germany). Three housekeeping genes were used: glyceraldehyde-3-phosphate dehydrogenase (GAPDH), β-actin (ACTB), and histone deacetylase 1 (HDAC1). The mean expression of these genes was used as the reference for normalizing target gene expression. Results are presented in arbitrary units as the ratio of target gene expression to the mean expression of the housekeeping genes.

#### 4.2.4. Statistical Analysis

Gene expression data were normalized to the mean relative mRNA level in the control group, which was set to 1.0. Statistical analyses were conducted on raw data after verifying the assumptions of normality using the Shapiro–Wilk test. Data that did not meet the normality criterion were logarithmically transformed before further analysis. Hormone concentrations, protein levels, and gene expression data were analyzed using one-way analysis of variance (ANOVA), followed by Tukey’s Honest Significant Difference (HSD) post hoc test. In the figures, different capital letters (A, B, C) denote statistically significant differences at *p* < 0.05, as determined by one-way ANOVA followed by Tukey’s HSD post hoc test. Groups sharing the same capital letter do not differ significantly, whereas groups labeled with different letters differ at a statistically significant level. All data are presented as mean ± SEM.

## 5. Conclusions

In summary, our study demonstrates that acute central administration of AEA prevents the inhibitory effect of inflammation on GnRH synthesis in the POA and even stimulates it. Inflammation significantly decreased GnRH concentration and gene expression in the POA, and centrally injected AEA reversed these inhibitory effects of LPS. This effect may result from both suppression of centrally synthesized proinflammatory IL-1β and stimulation of anti-inflammatory IL-10 synthesis. Furthermore, it seems that these changes may be partially due to the AEA-dependent reduction in HPA axis activity in LPS-treated animals. However, in the short term, central AEA administration did not counteract the inflammation-induced reduction in LH secretion. These findings support the role of endogenous cannabinoids in the regulation of reproductive processes at the CNS level. However, in order to better understand the mechanisms by which endocannabinoids modulate GnRH/LH secretion during immunological stress, further in-depth studies using central administration of specific CB1R and CB2R receptor antagonists are required. Moreover, our study examined the effect of a single ICV administration of AEA on the activity of the hypothalamic-pituitary unit. It seems that future studies on the influence of long-term administration of this compound are necessary.

It should be emphasized that sheep is one of the most recognized large animal models for research on the neuroendocrine regulation of reproductive processes as well as on immune-neuroendocrine interactions. Therefore, a better understanding of the role of the endocannabinoid system in regulating reproductive functions in the sheep model and the results obtained may contribute to the further exploration of cannabinoids for medical use.

## Figures and Tables

**Table 1:** Experimental groups.

Graph.	No. of Animal	Experimental Treatment I (IV)	Dose [ng/kg]	Experimental Treatment II (IV/ICV)	Dose [µM/Animal]
Control	6	NaCl	0	NaCl	0
LPS IV	6	LPS	400	NaCl (iv.)	0
LPS IV and AEA ICV	6	LPS	400	AEA (ICV)	100
Total	18				

**Table 2:** Primer sequences and target genes used for real-time PCR analysis.

GenBank Acc. No.	Gene	Amplicon Size [bp]	Forward/Reverse	Sequence 5′ → 3′	Ref.
NM_001034034	*GAPDH* *glyceraldehyde-3-phosphate dehydrogenase*	134	forward	AGAAGGCTGGGGCTCACT	[45]
			reverse	GGCATTGCTGACAATCTTGA	
U39357	*ACTB* *beta actin*	168	forward	CTTCCTTCCTGGGCATGG	[45]
			reverse	GGGCAGTGATCTCTTTCTGC	
BC108088.1	*HDAC1* histone deacetylase1	115	forward	CTGGGGACCTACGGGATATT	[35]
			reverse	GACATGACCGGCTTGAAAAT	
NM_001009397	*GnRHR* *gonadotropin-releasing hormone receptor*	150	forward	TCTTTGCTGGACCACAGTTAT	[45]
			reverse	GGCAGCTGAAGGTGAAAAAG	
U02517	*GnRH* *gonadotropin-releasing hormone*	123	forward	GCCCTGGAGGAAAGAGAAAT	[45]
			reverse	GAGGAGAATGGGACTGGTGA	
X52488	*LHB* *luteinizing hormone beta-subunit*	184	forward	AGATGCTCCAGGGACTGCT	[45]
			reverse	TGCTTCATGCTGAGGCAGTA	

## Data Availability

The original contributions presented in this study are included in the article. Further inquiries can be directed to the corresponding authors.
